# Preparation, loading, and cytotoxicity analysis of polymer nanotubes from an ethylene glycol dimethacrylate homopolymer in comparison to multi‐walled carbon nanotubes

**DOI:** 10.1002/jin2.7

**Published:** 2016-04-21

**Authors:** Ben Newland, Laurent Thomas, Yu Zheng, Martin Steinhart, Carsten Werner, Wenxin Wang

**Affiliations:** ^1^ Leibniz‐Institut für PolymerforschungDresdenGermany; ^2^ Brain Repair Group, School of BiosciencesCardiff UniversityCardiffUK; ^3^ The Charles Institute of Dermatology, School of Medicine and Medical ScienceUniversity College DublinDublinIreland; ^4^ Institut für Chemie neuer Materialien, Universität OsnabrückBarbarastraße 7Osnabrück49069Germany; ^5^ School of Materials Science and EngineeringTianjin UniversityTianjinChina

**Keywords:** Anodised aluminium oxide (AAO) template, C6 glioma, drug delivery, EGDMA, multi‐walled carbon nanotubes (MWCNT), polymer nanotubes

## Abstract

Despite concerns over toxicity, carbon nanotubes have been extensively investigated for potential applications in nanomedicine because of their small size, unique properties, and ability to carry cargo such as small molecules and nucleic acids. Herein, we show that polymer nanotubes can be synthesized quickly and easily from a homopolymer of ethylene glycol dimethacrylate (EGDMA). The nanotubes formed via photo‐initiated polymerization of the highly functional prepolymer, inside an anodized aluminium oxide template, have a regular structure and large internal pore and can be loaded with a fluorescent dye within minutes representing a simple alternative to multi‐walled carbon nanotubes for biomedical applications.

## Introduction

Until recently, obtaining soluble polymers via radical homopolymerization of multi‐vinyl monomers such as ethylene glycol dimethacrylate (EGDMA) remained a challenge. Typically, very low quantities (typically 1–3%) of such multi‐functional monomers had been used as a branching agent in copolymerization reactions (Bouhier, Cormack, Graham, & Sherrington, 2007; Wang, Li, Ryan, & Armes, 2006; Wang & Zhu, 2005). However, techniques such as deactivation‐enhanced atom transfer radical polymerization and reversible addition fragmentation chain transfer (RAFT) polymerization have allowed researchers to push the boundaries of copolymerization branching from 10% EGDMA inclusion (Newland et al., 2010; Newland et al., 2012; Newland et al., 2014), all the way to homopolymer of EGDMA (Zheng, Zhao, Newland, Poly, & Wang, 2013; Zheng et al., 2011). This has lead not only to higher degrees of branching but also to the formation of a new polymer structure consisting of single polymer chains looped to themselves via intramolecular cyclization reactions (Zheng, Newland, Tai, Pandit, & Wang, 2012). These polymer structures also contain a high percentage of free vinyl groups (i.e. not used for the chain growth or the intramolecular cyclizations).

The aim of this work was to assess whether these single cyclized knot polymers, with high vinyl functionality, could be used as a simple way to create polymer nanotubes. Nanoscale materials with a high aspect ratio, such as nanorods, nanowires and nanotubes, have been synthesized from a variety of different materials for applications ranging from electrical conductors (Abidian, Ludwig, Marzullo, Martin, & Kipke, 2009), enhancing fluid flow (Whitby, Cagnon, Thanou, & Quirke, 2008; Whitby & Quirke, 2007), fluorescence detection (Barone, Baik, Heller, & Strano, 2005; Lee, Müller, Al‐Kaysi, & Bardeen, 2006), and biomedical devices (Al‐Jamal et al., 2011; Hillebrenner, Buyukserin, Stewart, & Martin, 2006). Since an early report of carbon nanotubes (CNTs) (Iijima, 1991), much research has focused on the application of these graphitic materials for enhancing material strength. However, CNTs have also been proposed for biomedical applications such as drug delivery (Liu, Tabakman, Welsher, & Dai, 2009), bone tissue engineering (Gupta et al., 2013), nucleic acid delivery (Al‐Jamal et al., 2011), and directing the fate of stem cells (Chen & Hsiue, 2013). Aside from the commonly reported drawback of carbon nanotube toxicity (Kostarelos, 2008; Lanone, Andujar, Kermanizadeh, & Boczkowski, 2013), they often require surface modification for loading with an external cargo when used as a drug delivery device (Liu et al., 2011).

Polymer nanotubes may serve as a highly attractive alternative to CNTs if they can be proven to be less toxic, perhaps degradable and provide a simple means to load drugs or small molecules within the pore of the nanotube without functionalization. Herein, we investigate whether a homo‐polymer of EGDMA with a high content of free vinyl groups, synthesized by RAFT polymerization, could be crosslinked within a porous anodized aluminium oxide (AAO) sacrificial template. AAO filters with aligned and highly regular pore structures have previously been used to form carbon “nanopipes” via carbon vapour deposition (Whitby et al., 2008), or polymer nanotubes, either by melt wetting (Song, She, Fu, & Li, 2004), or by immobilizing initiator groups or a catalyst within the pores and growing polymer chains via living polymerization methods (Cui et al., 2005; Jang, Ko, & Kim, 2006). In contrast, our objective was to synthesize a prepolymer, which could be well characterized prior to nanotube formation via photo‐polymerization. Finally, it was desired to analyse whether nanotubes formed by this method could be loaded with small molecules to represent the possibility for drug delivery applications.

## Materials and Methods

### Materials

The monomer ethylene glycol dimethacrylate (EGDMA), the initiator 1,1′‐azobis (cyclohexanecarbonitrile) (ACHN), and the chain transfer agent 2‐Cyanopropan‐2‐yl benzodithioate (CPBD) were purchased from Sigma‐Aldrich (Sigma‐Aldrich, Munchen, Germany). The solvent toluene (HPLC grade, Sigma‐Aldrich) and precipitation agent n‐hexane (ACS reagent grade, Sigma‐Aldrich) were used as received. Anodized aluminium oxide porous membranes with an inner pore diameter of ~200 nm (Whatman, Maidstone, UK) were purchased from Sigma‐Aldrich. The photo‐initiator (2‐hydroy‐2‐methylpropiophenone) and dye (rhodamine 6G) were also purchased from Sigma‐Aldrich.

### Homopolymerization of ethylene glycol dimethacrylate

The EGDMA homopolymer was synthesized as reported previously (Zheng et al., 2012). Briefly, 110 mg of CPBD was dissolved in 2 mL of toluene and added into a 100‐mL round bottomed flask. Then a further 18 mL of toluene was added, before the addition of 9.9 g of EGDMA. The flask was sealed, and oxygen was removed by bubbling argon through the solution for 15 min in an ice bath. Then 48.8 mg of ACHN was added to the flask, and argon was bubbled through for another 5 min. The mole ratio of ACHN : CPBD : EGDMA was 0.4:1:100. The flask was then placed in an oil bath preheated to 70°C with stirring at 700 rpm. The reaction proceeded for 4 h before being stopped by the addition of air. Purification was carried out by simply diluting the polymer in acetone followed by precipitation in a large excess of cold hexane to remove the EGDMA monomer. The pink coloured precipitate was filtered using filter paper (Whatman, Qualitative‐1) before being dried by vacuum evaporation at room temperature for 6 h. The final yield was obtained by weighing the dry polymer powder.

### Polymer characterization

Characterization of the knot polymer was carried out using gel permeation chromatography equipped with a refractive index detector (Varian 920‐LC (Palo Alto, CA, USA)). Number average molecular weight (Mn), weight average molecular weight (Mw), and polydispersity (Mw/Mn) were obtained via two 30‐cm PLgel Mixed‐C in series calibrated with PMMA standards (maximum parsimony from 690 to 1,944,000 g/mol) using dimethylformamide (DMF) as an eluent (Zheng et al., 2012). ^1^H NMR analysis was carried out in deuterated chloroform using a 300‐MHz Bruker NMR with MestReC processing software (Billerica, MA, USA).

### Nanotube synthesis

The prepolymer solution for the synthesis of the polymer nanotubes was prepared by diluting the EGDMA homopolymer in acetone to a concentration of 5 wt./vol.% before adding the liquid photo‐initiator at 0.1 wt./wt.% (of polymer). A 5‐μL drop of this prepolymer solution was then added on top of the AAO template, whereupon it quickly spreads but was assisted by moving the pipette tip until all of the AAO template was covered (it had a slight pink colour because of the pink colour of the prepolymer solution). The template was then transferred directly under UV light (Delo (Windach, Germany), Delolux 04, 315–500 nm, with an intensity of 8000 mW/cm^2^), for 1 min to photo‐crosslink the prepolymer into the nanotube shape within the pores. The nanotubes were removed from the template by the addition of 1‐mol sodium hydroxide to the template and leaving it submerged for 30 min, vortexing when necessary. The nanotubes were then washed twice with sodium hydroxide, centrifuging at 13,000 rpm between washes to collect the pellet. The nanotubes were then washed a further two times in ethanol and dried under a sterile work bench if required for toxicity studies.

### Nanotube characterization

Because of the large lengths of the nanotubes, the length characterization was performed by light microscopy using a 40x lens (Zeiss – Axio Observer.Z1 (Oberkochen, Germany)). A suspension of nanotubes in ethanol was diluted until individual nanotubes could be observed on a microscope slide with little or no overlap (see representative Fig. 4). Because the nanotubes did not aggregate, this was not difficult. The nanotubes were allowed to dry, and five images were taken; a total of 170 nanotubes were measured using ImageJ (NIH, Bethesda, MD, USA) software. For scanning electron microscopy analysis, nanotubes were suspended in ethanol at a concentration of 1 mg/mL, and drops were added to a microscope slide and allowed to dry in a vacuum oven at 40°C under reduced pressure (to ensure the pore is fully dried) for 2 h. They were then scraped with a razor blade onto a preprepared scanning electron microscope (SEM) stub and sputter coated with gold (SCD 050, BAL‐TEC, Witten, Germany) for 60 s. Imaging was performed with a Philips Environmental SEM (XL30) (Amsterdam, Netherlands) but in SEM mode with a beam intensity of 25 kV.

### Cell culture

C6 rat glioma cells were purchased from Sigma‐Aldrich and cultured in Dulbecco's modified eagle's medium (Gibco) containing 10% fetal bovine serum and 1% penicillin and streptomycin in a humidified atmosphere containing 5% CO_2_ at 37°C as reported elsewhere (Han, Xu, Li, Ren, & Yang, 2012). Primary astrocytes previously extracted from the new‐born rat pups (Newland et al., 2013) were cultured in Dulbecco's modified eagle's medium supplemented with F12 Ham mixture (50%) (Sigma‐Aldrich), 10% fetal bovine serum, and 1% penicillin and streptomycin. Cells were seeded in a 96 well‐plate (10,000 cells/well for astrocytes and 5000 cells/well for C6 cells) and left for 24 h to attach and grow prior to addition of the nanotubes.

### Cytotoxicity analysis

Polymer nanotubes and multi‐walled carbon nanotubes (MWCNTs) (Sigma‐Aldrich) were suspended in cell culture media (containing serum) at various concentrations. The media were removed from the seeded well‐plates (C6 glioma and astrocytes), and 100 μL of the various dilutions was added and incubated for 24 h. The cells were then washed with phosphate buffered saline, and new media were added that contained 10% PrestoBlue® cell viability (Invitrogen, Thermo Fisher Scientific, Germany) reagent. This PrestoBlue® solution was also added to empty wells to act as a blank. The cells were incubated for 30 min in PrestoBlue® solution, and the fluorescence intensity of the resulting resazurin product was measured in a plate reader (TECAN, Männedorf, Switzerland) and analysed according the manufacturer's protocol. Experiments were performed in quadruplet with cells receiving media containing no nanotubes as a negative control normalized as 100% viable.

### Loading with fluorescent dye

A stock solution of Rhodamine 6G (Sigma‐Aldrich) at 280 µg/mL in distilled water was prepared. Two hundred microliters of the rhodamine stock was added to 300 μL of a stock of polymer nanotubes (300 µg/mL in distilled water) (final Rhodamine concentration 112 µg/mL – final tube concentration 180 µg/mL). The mixture was vortexed for a few seconds then centrifugated (13.4 rpm – 4 min). The supernatant was removed, and the pellet was resuspended in 500 μL of distilled water. After three washes (in water), 1 μL of the labelled nanotubes was dropped onto a glass slide. As a control, 1 μL of nonloaded nanotubes (180 µg/mL) was dropped on the same glass slide. A fluorescence microscope (Zeiss – Axio Observer.Z1 – excitation wavelength 530 nm, emission wavelength 552 nm) was used to image the nanotubes using a 20x lens, and Zen Software (Rochdale, UK) (2012 Version 1.1.2.0) was used to process the images.

## Results and Discussion

**Figure 1:**
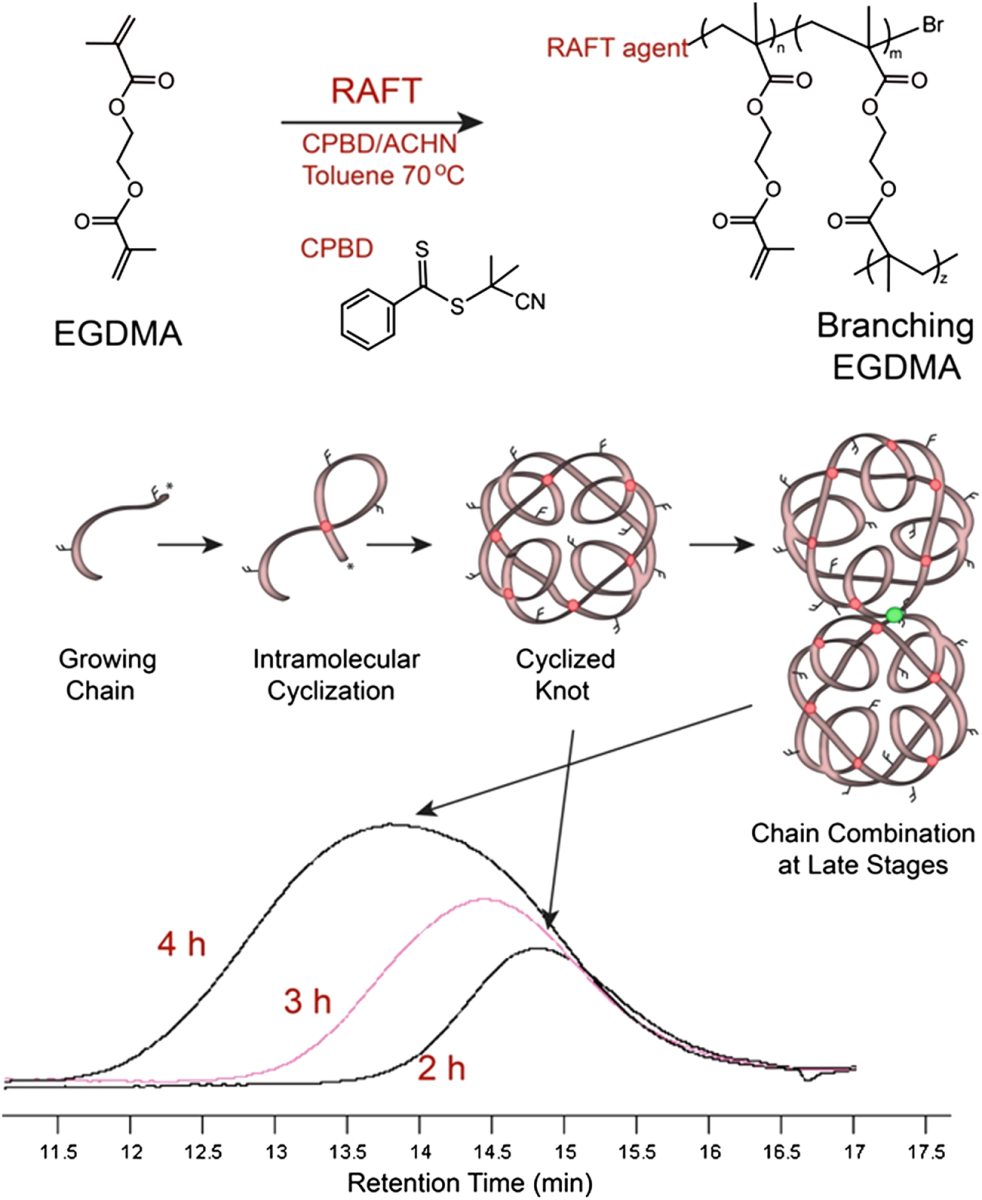
A schematic representation of the homopolymerization of ethylene glycol dimethacrylate (EGDMA) via reversible addition fragmentation chain transfer (RAFT) polymerization, depicting the intramolecular cyclization occurring. The gel permeation chromatogram is shown for samples taken during the reaction, showing phase 1 where a low polydispersity index and highly symmetrical peaks (2 and 3 h) indicate linear chain growth (and cyclization) before chain combination at later stages (4 h).

Figure 1 shows the RAFT polymerization procedure with a schematic diagram of the growth of the intramolecular crosslinked chains from the monomer EGDMA. The deactivating nature of the reaction suppresses intermolecular crosslinking (traditional hyperbrancing by chain combination) and allows polymer chains to grow via propagation reactions and internal cyclization reactions. This can be visualized by the highly symmetrical peaks in the gel permeation chromatography chromatogram (Fig. 1) for the early time points (Zheng et al., 2012). Only at later reaction stages did the chains combine to form multi‐knot structures (Aied, Zheng, Newland, & Wang, 2014). This can be seen via a broadening of the peak and a rapid increase in the polydispersity index (*M_w_*/*M_n_*)(*Đ*). After 4 h of reaction time, the final molecular weight of the polymer after purification was 12,600 (*M_n_*), 29,700 (*M_w_*) with *Đ =* 2.36, and a monomer conversion of 48%. ^1^H NMR spectroscopy analysis (Figure S1) revealed free vinyl groups within the structure, which were essential for subsequent crosslinking of the prepolymer into the nanotube structures.

**Figure 2:**
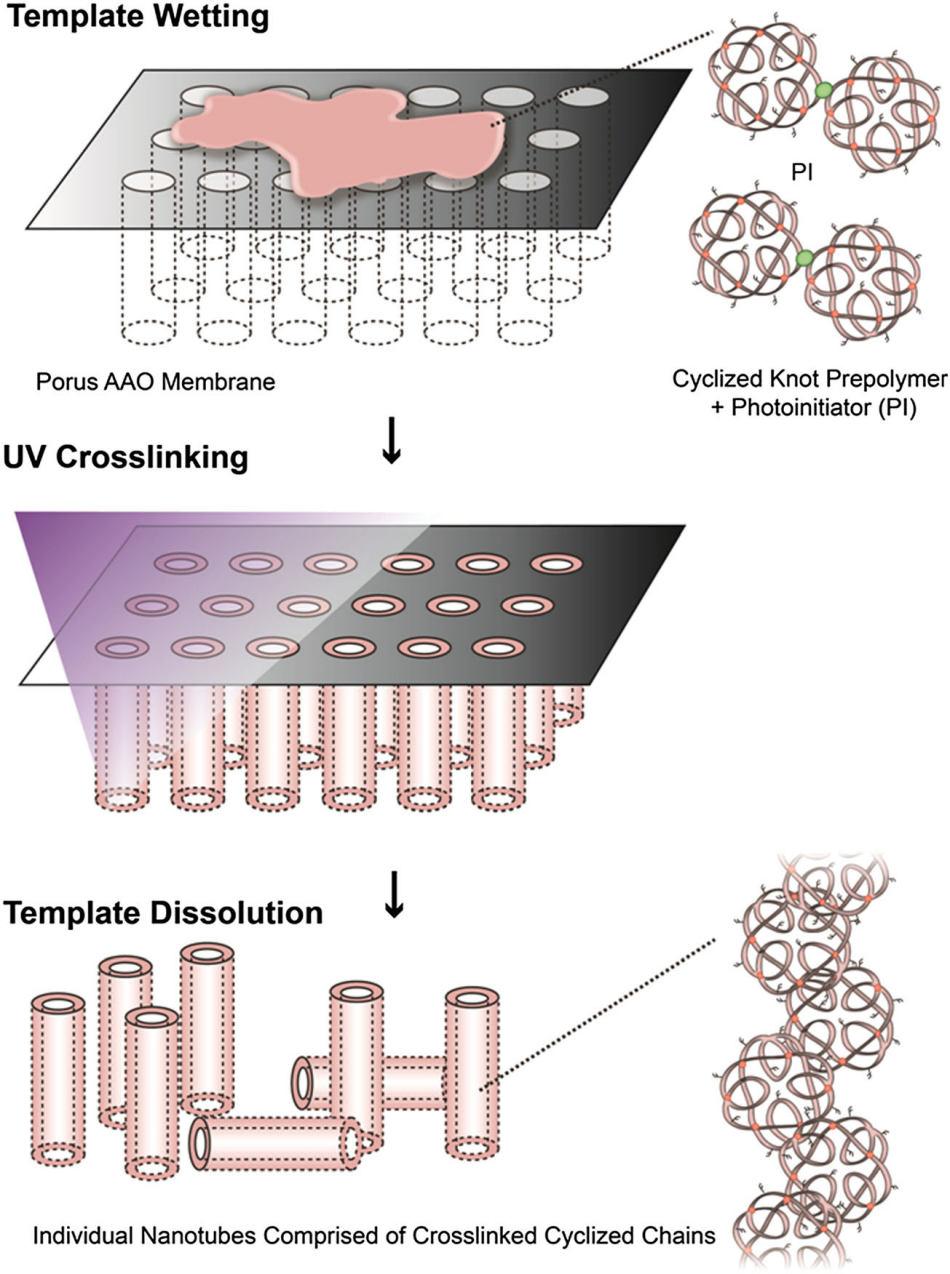
A schematic depiction of the formation of polymer nanotubes via wetting of the porous AAO template with the prepolymer and photo‐initiator followed by UV crosslinking for one minute. Template dissolution in sodium hydroxide reveals free tubes of the crosslinked polymer.

**Figure 3:**
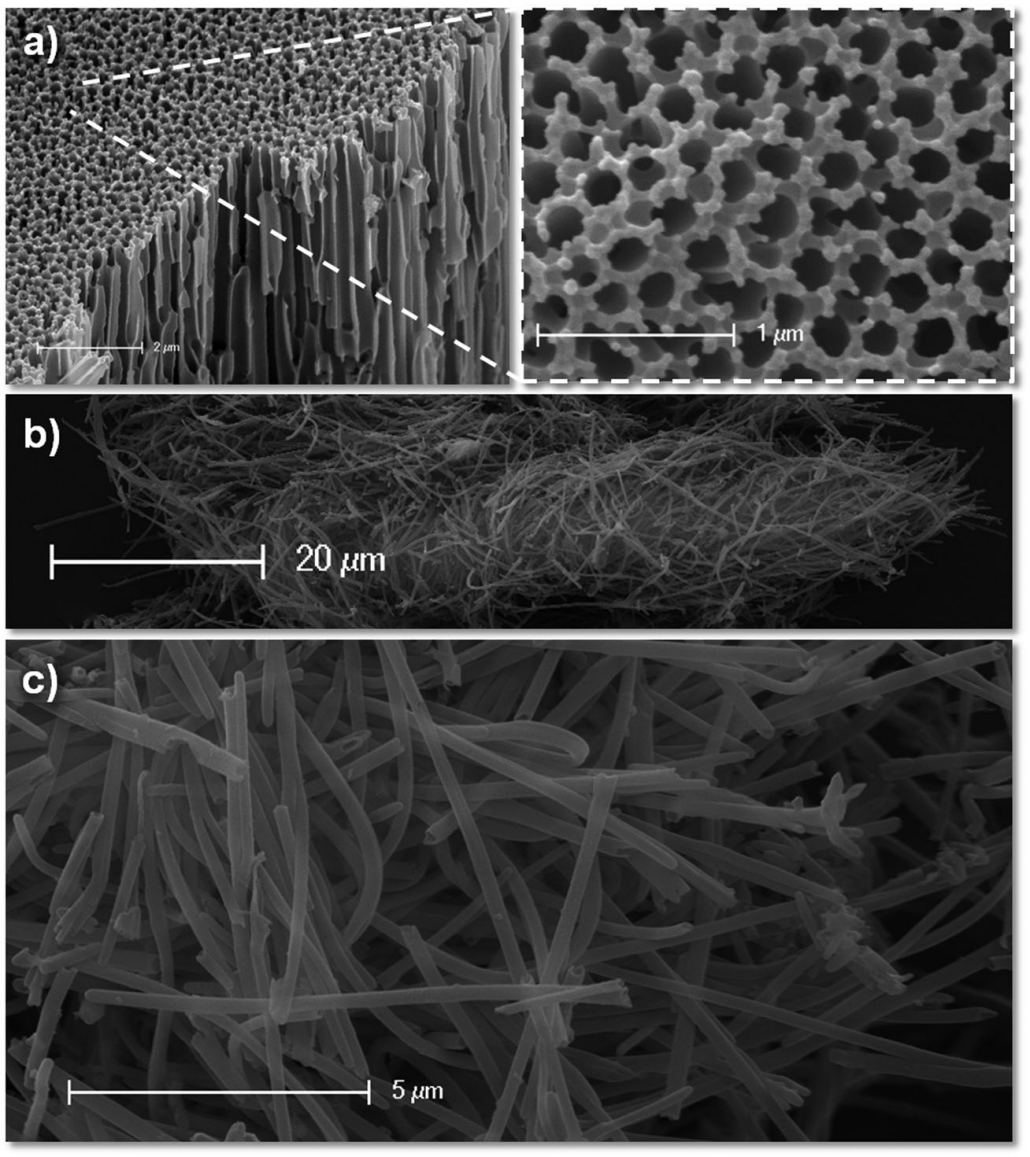
Scanning electron microscopy of the AAO template (A), showing the highly regular nature of the pores. Large quantities of nanotubes can be obtained (B) without signs of debris material (B, C).

Anodized aluminium oxide porous filters (Whatman) were used as a template in which to photo‐crosslink the prepolymer (see Fig. 2 for a schematic representation of nanotube formation). Although nanotubes could be formed just by wetting the template with the prepolymer solution and exposing it to UV light for 1 min, the yield was not good, and only short lengths of tubes could be observed amongst much unstructured debris like material. Once the photo‐initiator was added (at 1 wt./vol.%), large yields with no debris material could be formed. This greater length was almost certainly due to better crosslinking of the prepolymer by a radical reaction occurring down the pore where UV light penetration was probably poor. The highly uniform nature of the pores of such filter membranes (Fig. 3A) with narrow pore walls (i.e. no wasted space) allows large quantities of nanotubes to be synthesized with no impurities (Fig. 3B and C). In principle, such membranes could be used for the formation of crosslinked polymer rods; however, in this study, we used a dilute solution of prepolymer in the highly volatile solvent acetone. In this way, the solvent evaporates quickly, leaving the prepolymer coating the pore wall prior to crosslinking, and thus allows the formation of hollow nanotubes. The dissolution of the AAO template with NaOH appears not to affect the nanotube formation despite EGDMA containing ester groups, which are typically susceptible to ester hydrolysis (Zhang et al., 2014). However, the minimum time possible for template dissolution is used (30 min or less) to reduce the risk of degrading the nanotubes.

**Figure 4:**
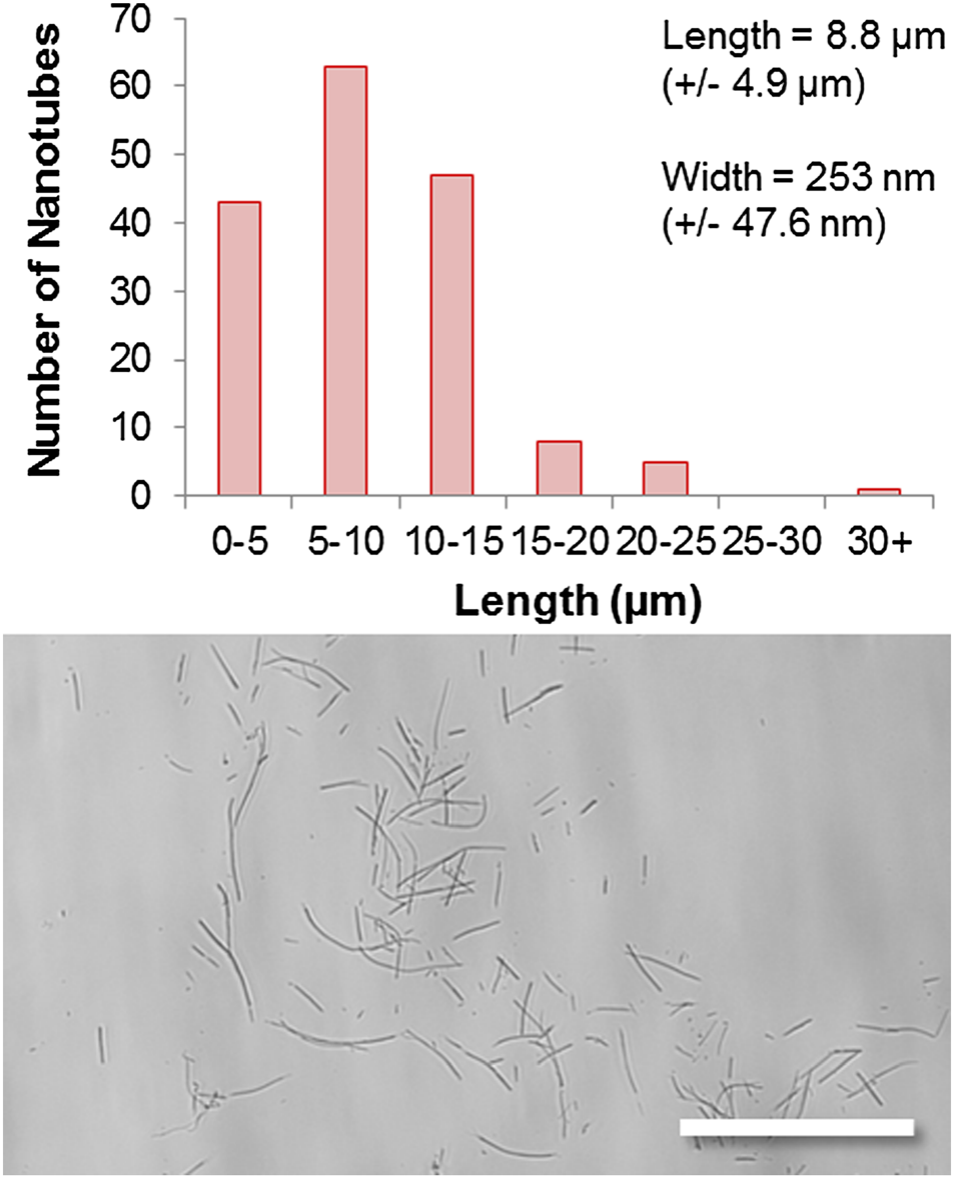
Characterization of the polymer nanotubes was carried out by scanning electron microscopy and light microscopy, showing an average length of 8.8 µm and an average width of 253 nm, with an example image below (scale bar = 50 µm).

The nanotubes have an average length of 8.8 µm (+/−4.9 µm) and an average width of 253 nm (+/−47.6 nm) with the majority of the nanotubes being 5–10 µm in length (Fig. 4). Indeed, the width appears (by observation only) to be much more regular for the polymer nanotubes than MWCNT that were used as a comparison control for the cytotoxicity experiments (see Figure S2 to see the large width deviation and presence of impurities). The reported depth of the AAO template is 60 µm according to the manufacturer, so theoretically, it should be possible to make even longer nanotubes; however, as the aspect ratio may affect toxicity (Kostarelos, 2008; Marchesan, Kostarelos, Bianco, & Prato, 2015), longer nanotubes may not necessarily be better for applications in nanomedicine. It would also be interesting to investigate the formation of nanotubes in AAO membranes that have a defined pore depth, such as 600 nm or 1 µm as reported elsewhere for nanorod array formation (Grimm et al., 2010).

**Figure 5:**
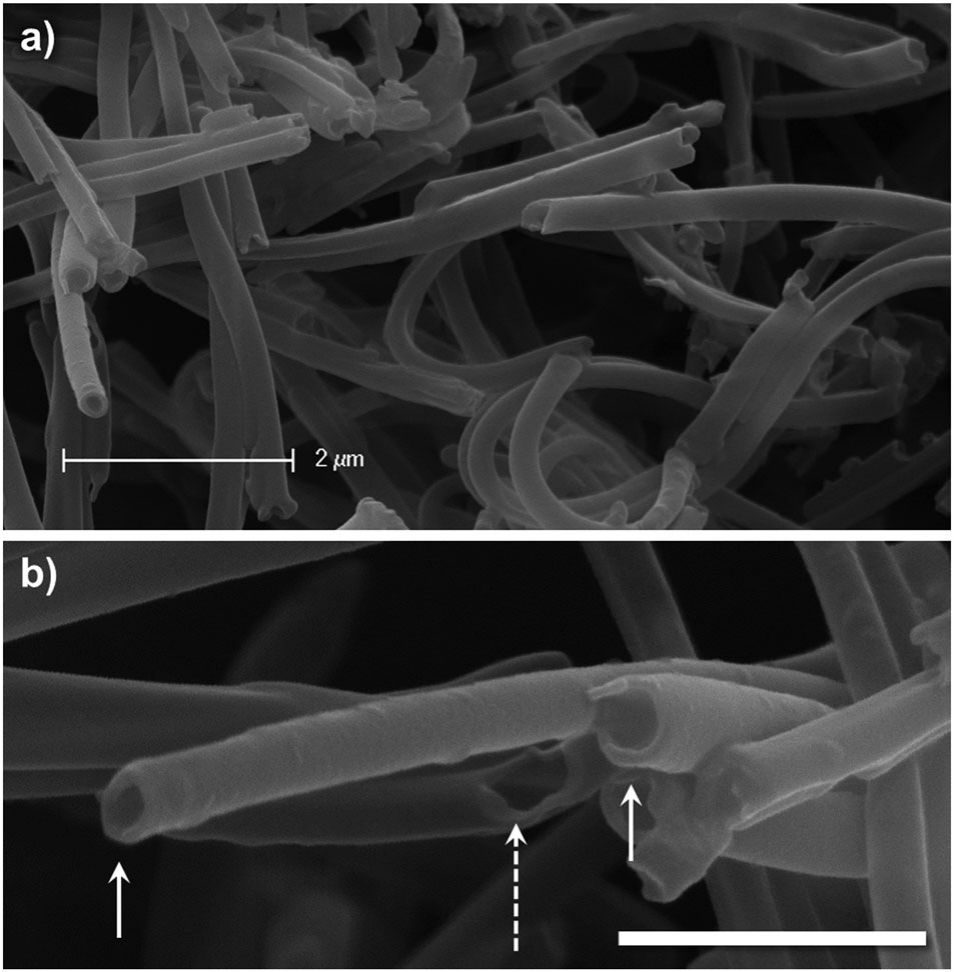
High magnification scanning electron microscopy images of the polymer nanotube ends allow the visualization of the pores (examples marked with arrows) and even a tear/defect in the nanotube wall (dashed arrow) to reveal the inside of the pore (lower scale bar = 1 µm).

Scanning electron microscopy was also used to carefully observe the nanotube ends where the large pore opening can clearly be observed (Fig. 5). It is important to note here that drying in the vacuum oven was critical for good sample preparation as tubes just dried by evaporation of the ethanol media still seemed to remain filled with some solution, visible by a cloud of elections gathering at the tube ends (data not shown). The long length of these polymer nanotubes, together with the large pore opening, provides rationale for assessing their use as a drug delivery device. The interesting dimensions of these nanotubes (large for nanotubes and small for sustained delivery devices) may make them ideal for extracellular drug delivery over extended periods if the drug can be loaded within the pore (Newland et al., 2015). The dashed arrow in Figure 5B shows a tube wall defect where it can be seen as “ripped open” at a bend point. Such defects were rare (only one observed), which is probably due to the seeming flexible nature of the nanotubes. Indeed, an interesting observation was made during microscopy analysis that the beam strength with an accelerating voltage of 25 kV was enough to cause the extension or movement of the nanotubes over the space of one minute (Figure S3).

**Figure 6:**
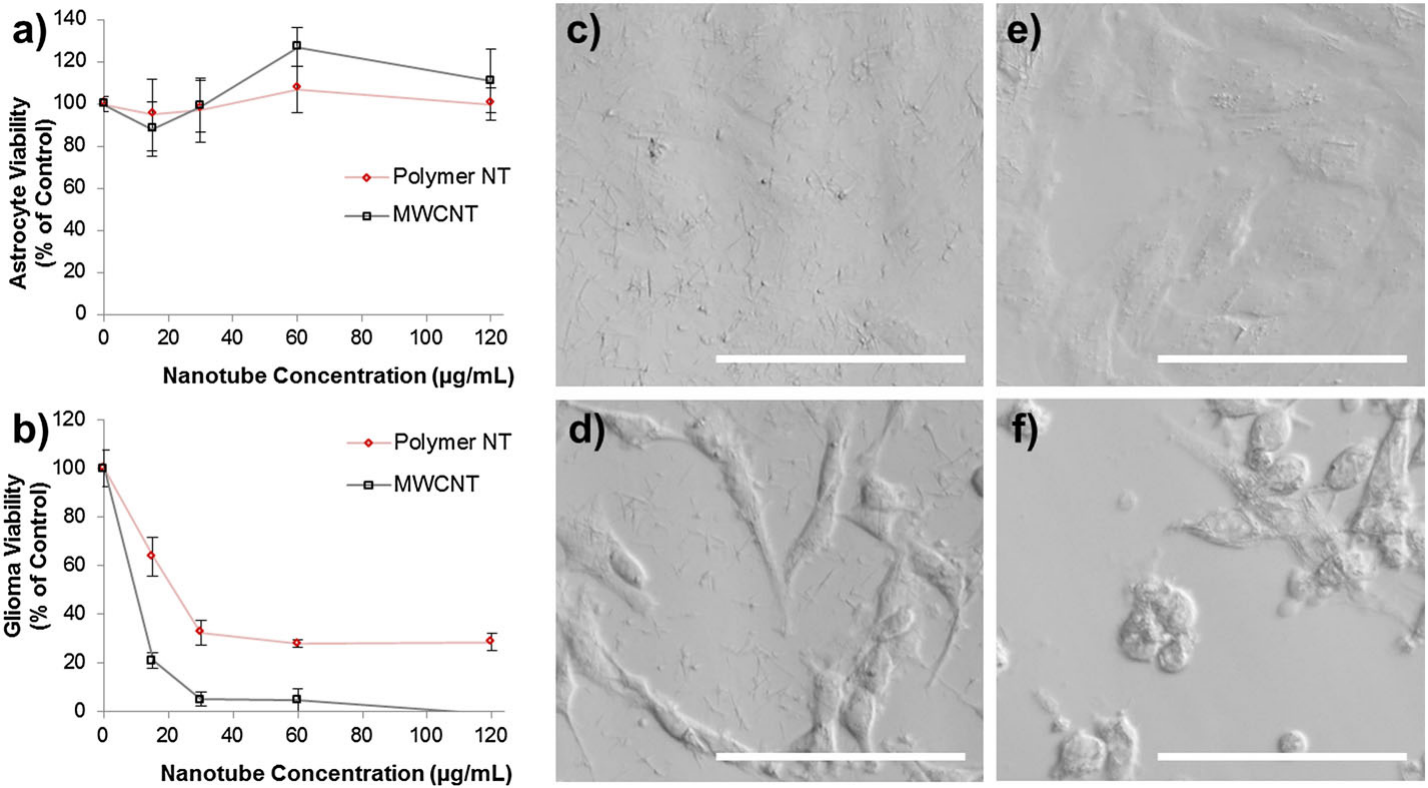
Cytotoxicity analysis of the polymer nanotubes in comparison with multi‐walled carbon nanotubes (MWCNT) when incubated for 24 h on astrocytes (A) and C6 glioma cells (B) showing that polymer nanotubes exhibited lower toxicity than MWCNT for the C6 glioma, but not for astrocytes where both nanomaterials were tolerated at the concentrations tested (*n* = 4, error bars represent +/−standard deviation). Light microscopy after 4 h of incubation with the polymer nanotubes (60 µg/mL) shows an even coverage across the well bottom of both astrocytes (C) and C6 glioma cells (D); however after 24 h and three washes with phosphate buffered saline, the astrocytes are largely devoid of nanotubes (E), yet C6 glioma cells have many nanotubes associated with their membrane (multiple thin parallel lines). And many are rounded or washed off the plate completely (F). Scale bars represent 100 µm.

The toxicity of the nanotubes was assessed using two cell types. Primary astrocytes extracted from the new born rat cortex and the cell line rat C6 glioma. Because future applications of these nanotubes for anti‐cancer drug deliver will be investigated, the C6 glioma was chosen along with a predominant cell type of the healthy adult brain: astrocytes. It should be noted here that the primary astrocyte cultures contained no accompanying microglia from the extraction process because it has been shown that the region of extraction can change the observed toxicity of carbon nanotubes depending whether the extraction region was poor or rich with microglia (Bussy et al., 2015). The toxicity of the nanotubes was assessed in comparison with MWCNTs obtained commercially without further purification. Figure 6 shows the differential toxicity towards the different cell types, with a remarkable toleration of the nanotubes by the astrocytes. In contrast, the C6 glioma, with a far greater proliferation rate, showed effects of the nanotubes at concentrations as low as 15 µg/mL. Lower toxicity was generally observed for the polymer nanotubes for all concentrations tested compared with the MWCNT. Microscopy analysis of the polymer nanotubes after 4 h of incubation shows an even coverage across both cell types. However, after 24 h, the cells were washed three times with phosphate buffered saline to remove free nanotubes, and subsequent analysis showed extensive association of the nanotubes with C6 glioma (Fig. 6D and F) but not with the astrocytes (Fig. 6C and E). This may well explain the differential toxicity between the two cell types tested. The washing not only allowed the visualization of the interaction between cell and nanomaterial but also reduced the effect MWCNT could have on the PrestoBlue assay (Figure S4). The comparative toxicity of the polymer nanotubes and the MWCNT requires further extensive investigation to determine the method of cell death. Future studies will investigate whether cell death occurs predominantly through necrosis or apoptosis and the cell membrane integrity will be analysed. However, this data suggest that the toxicity of the polymer nanotubes is lower than MWCNT but is highly cell type dependent. Another interesting possibility for reducing the toxicity is to engineer the nanotubes to be biodegradable. Such work is already underway for carbon nanotubes (Nunes et al., 2012; Sureshbabu et al., 2015), but for polymer nanotubes, this could be simply introduced into the polymer structure by use of monomer containing a disulfide bond and synthesizing a hyperbranched polymer instead of a knot polymer structure (Zhao et al., 2014).

**Figure 7:**
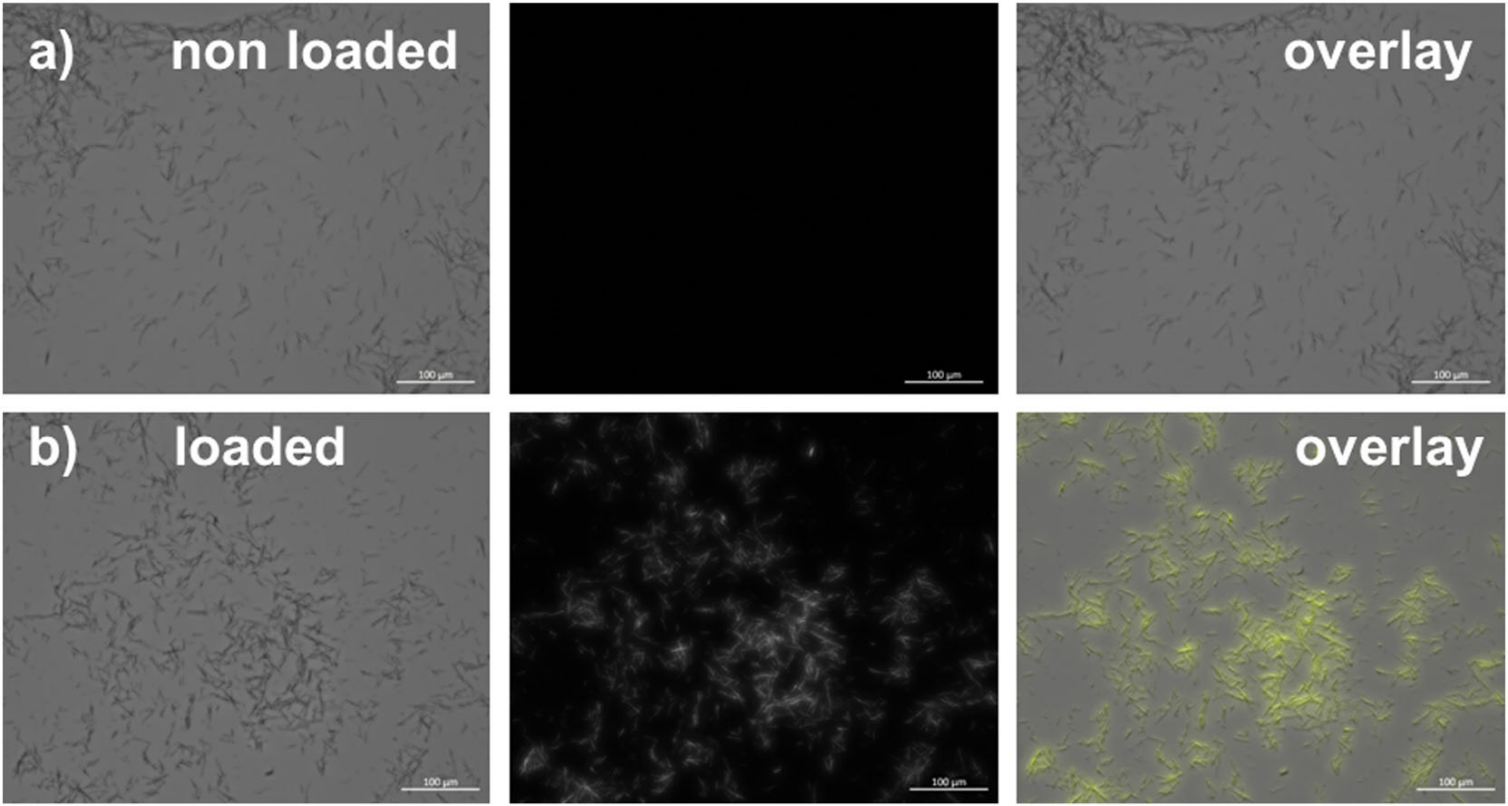
A proof of principle that the polymer nanotubes could be used for small molecule delivery. Polymer nanotubes alone (top panel) or loaded with the fluorescent dye rhodamine 6G (bottom panel) were investigated using fluorescence microscopy to show that the nanotubes could be loaded after 1 min of incubation with a rhodamine in water solution.

Finally, it was desired to analyse whether these polymer nanotubes could be loaded with a small molecule to prove the concept for future drug delivery applications. Rhodamine 6G was used as an example small molecule because of its fluorescence properties allowing its detection within/on the nanotubes. Figure 7A shows how the polymer nanotubes show no intrinsic fluorescent properties at excitation with light of wavelength 530 nm, whereas polymer nanotubes loaded with rhodamine 6G show a fluorescent glow clearly visible by fluorescence microscopy (Fig. 7B). Nonfunctionalized MWCNTs showed no such loading capability (data not shown) and have instead been shown to be loaded with cargo following noncovalent modifications such as with amphiphilic diblock copolymers (Di Crescenzo, Velluto, Hubbell, & Fontana, 2011), or covalent modifications such as EDC/NHS mediated linkage of amine containing polymers (Liu et al., 2011). Such a simple filling procedure for these EGDMA‐based polymer nanotubes, taking only a matter of minutes (including purification) exemplifies, the potential such polymer nanotubes hold for further biomedical applications.

## Conclusions

This study shows that polymer nanotubes can be formed from a highly functional prepolymer based on ethylene glycol dimethacrylate. RAFT polymerization was used to form the prepolymer, which allowed high control over the polymer molecular weight and structure. Characterization of the polymer could be carried out first, before proceeding with the nanotube synthesis step. The free‐vinyl content of the knot‐structured prepolymer allowed photo‐initiated crosslinking within a sacrificial AAO template, and free nanotubes could be released after dissolution of the template. The nanotubes have a highly ordered structure with a wide pore end, and in contrast to MWCNT, no debris was observed during any of the microscopy (light and electron). The nanotubes showed similar or lower toxicity profile than MWCNT and furthermore allowed uptake of the fluorescent dye rhodamine 6G to prove the concept that such nanotubes could be used for small molecule delivery. Future studies will assess the nanotube ability to deliver doxorubicin to cancer cell types in vitro. RAFT polymerization allows the inclusion of a wide range of commercially available monomers, so further investigations will continue with copolymerization systems for a variety of charge densities and functionalities.

## Supporting information


**Supplementary Figure S1** – ^1^H NMR spectrum of the EGDMA Pre‐polymer after purification with corresponding peak allocation to the chemical structure. The free vinyl groups in the polymer structure are clearly shown as those shifted to the left (a and b).
**Supplementary Figure S2** – showing two SEM images of the polymer nanotubes after intial exposure (top) to the electron beam, and after 1 minute without moving the stage (middle). An overly of the two images allows the visualization of the movement, with two example movements being highlighted with dashed arrows.
**Supplementary Figure S3** – An example SEM image of the MWNT showing the variety of widths of the MWNTs and the presence of debris material (non‐rod‐shaped).
**Supplementary Figure S4** – Incubation of the nanomaterials with the PrestoBlue assay for 1 hour to find out if there is inhibition or enhancement of the fluorescence signal. This data shows the maximum error of freely dispersed MWCNTs to be 18% simply due to interaction with the assay. No interaction could be observed for polymer nanotubes. However, the obtained data cannot be used to normalize the cytotoxicity data since the cells were washed three times prior to assessment which removed free‐floating nanotubes, so the real degree of error is likely to be much less for the cytotoxicity experiments.

Supporting info item
