# Supplementary material for: Preparation, loading, and cytotoxicity analysis of polymer nanotubes from an ethylene glycol dimethacrylate homopolymer in comparison to multi‐walled carbon nanotubes
Source: J Interdiscip Nanomed. 2016 Apr 21;1(1):9–18. doi: 10.1002/jin2.7 (PMC4959090; doi:10.1002/jin2.7)
Supplement: Supplementary file 1 — Supporting info item [file JIN2-1-9-s001.doc]

Preparation, loading and cytotoxicity analysis of polymer nanotubes from an ethylene glycol dimethacrylate homopolymer in comparison to multi-walled carbon nanotubes.

Ben Newland^1,2^*, Laurent Thomas^1^, Yu Zheng^3^, Martin Steinhart^4^, Carsten Werner^1^, Wenxin Wang^3,5^

^1^Leibniz-Institut für Polymerforschung, Dresden, Germany; ^2^Brain Repair Group, School of Biosciences, Cardiff University, Cardiff, UK; ^3^The Charles Institute of Dermatology, School of Medicine and Medical Science, University College Dublin, Dublin, Ireland; ^4^Institut für Chemie neuer Materialien, Universität Osnabrück, Barbarastraße 7, 49069 Osnabrück, Germany; ^5^School of Materials Science and Engineering, Tianjin University, Tianjin, China.

**Supplementary Information**


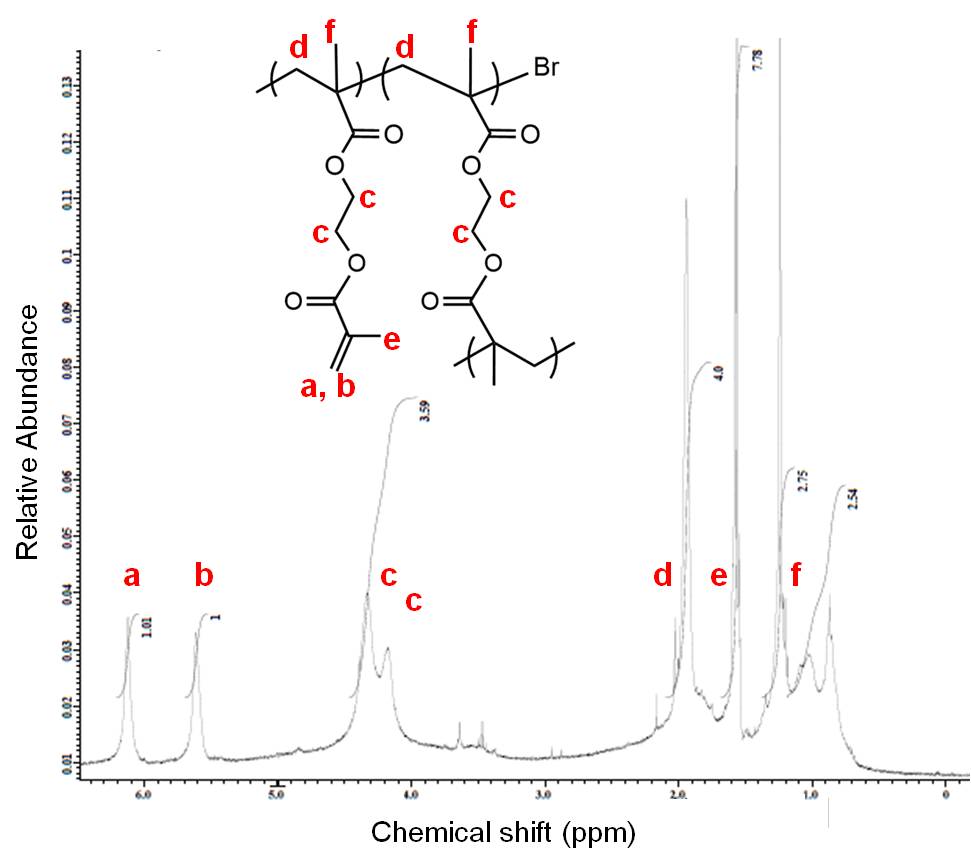


**Supplementary Figure S1 –** *^1^H NMR spectrum of the EGDMA pre-polymer after purification with corresponding peak allocation to the chemical structure. The free vinyl groups in the polymer structure are clearly shown as those shifted to the left (a and b).*

**
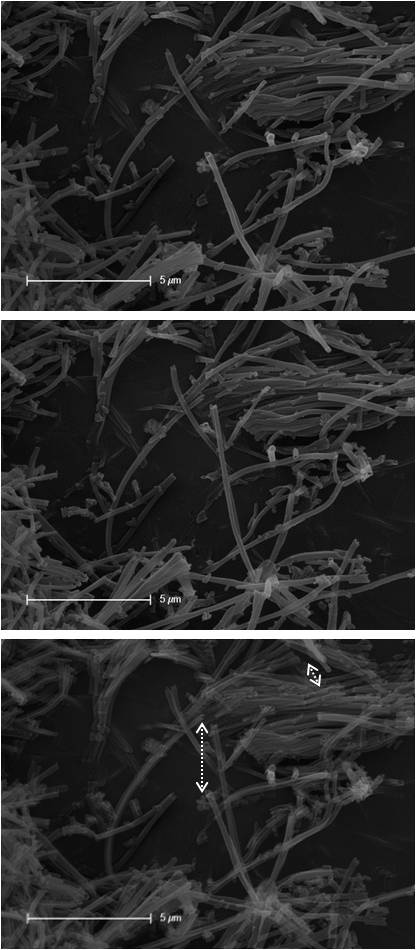
**

**Supplementary Figure S2 *–*** *showing two SEM images of the polymer nanotubes after intial exposure (top) to the electron beam, and after 1 minute without moving the stage (middle). An overly of the two images allows the visualization of the movement, with two example movements being highlighted with dashed arrows.*

*
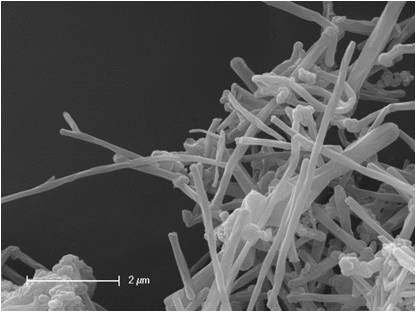
*

**Supplementary Figure S3** – *An example SEM image of the MWNT showing the variety of widths of the MWNTs and the presence of debris material (non-rod-shaped).*

*
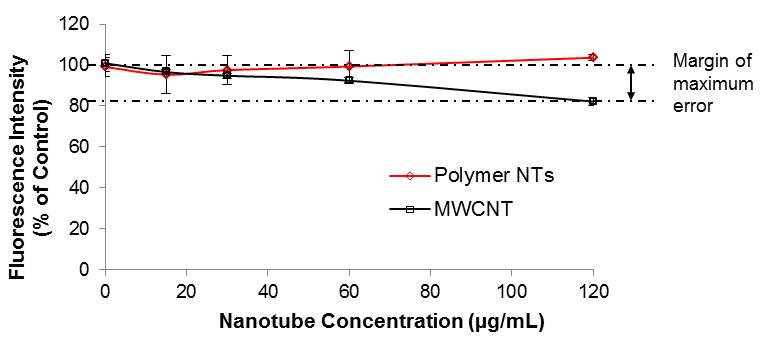
*

**Supplementary Figure S4** – *Incubation of the nanomaterials with the PrestoBlue assay for 1 hour to find out if there is inhibition or enhancement of the fluorescence signal. This data shows the maximum error of freely dispersed MWCNTs to be 18% simply due to interaction with the assay. No interaction could be observed for polymer nanotubes. However, the obtained data cannot be used to normalize the cytotoxicity data since the cells were washed three times prior to assessment which removed free-floating nanotubes, so the real degree of error is likely to be much less for the cytotoxicity experiments.*
